# Successful anesthetic management of >10-liter blood loss in a Budd-Chiari syndrome patient undergoing living donor liver transplantation: A case report and review of literature

**DOI:** 10.12669/pjms.42.1.13946

**Published:** 2026-01

**Authors:** Shahbaz Hussain, Yasir Bashir Butt, Salman Shahzad, Farooq Afzal, Eitzaz Ud Din Khan

**Affiliations:** 1 Shahbaz Hussain, Department of Anesthesia, Pakistan Kidney and Liver Institute, Research Center (PKLI & RC), Lahore, Pakistan; 2 Yasir Bashir Butt, Department of Anesthesia, Pakistan Kidney and Liver Institute, Research Center (PKLI & RC), Lahore, Pakistan; 3 Salman Shahzad, Department of Anesthesia, Pakistan Kidney and Liver Institute, Research Center (PKLI & RC), Lahore, Pakistan; 4 Farooq Afzal, Department of Anesthesia, Pakistan Kidney and Liver Institute, Research Center (PKLI & RC), Lahore, Pakistan; 5 Eitzaz Ud Din Khan, Department of Anesthesia, Pakistan Kidney and Liver Institute, Research Center (PKLI & RC), Lahore, Pakistan

**Keywords:** Anesthetic management, Budd-Chiari syndrome, Blood loss, Case report, Literature review, Living donor liver transplantation

## Abstract

**Background & Objective:**

Budd-Chiari syndrome is an uncommon condition induced by thrombotic or nonthrombotic obstruction of the hepatic venous outflow and is characterized by hepatomegaly, ascites, abdominal pain. The successful anaesthetic and transfusion treatment of a patient with BCS who had a hepatic hydatid cyst following LDLT is described in this report. Venous obstruction and parasitic cysts together pose a significant perioperative risk, especially in cases of severe intraoperative bleeding.

**Case Presentation:**

A 30 years old woman was admitted with weight loss, abdominal distension, recurrent haematemesis, and progressive jaundice. She had undergone multiple endoscopic variceal band ligations and treatment for pulmonary tuberculosis. Imaging revealed hepatic vein thrombosis, caudate lobe hypertrophy, and a hydatid cyst in segment VII that measured 5.8 × 4 cm. Anaemia (haemoglobin 7.6 g/dL) with preserved renal and coagulation function was found in the laboratory (MELD 14, Child-Turcotte-Pugh A6). LDLT was scheduled for her following multidisciplinary optimization.

**Management and Outcome:**

General anesthesia was achieved and then transesophageal echocardiography, arterial, central venous and PiCCO lines, were placed for invasive haemodynamic monitoring. The more than 10 liters of intraoperative blood loss were controlled by an organized massive transfusion protocol that used packed red blood cells, plasma, cryoprecipitate, and 2.9 litres of autologous blood through cell salvage. In order to preserve haemodynamic stability and keep mean arterial pressure above 70 mmHg, norepinephrine and vasopressin infusion were used. The patient showed stable graft function and recovered without any problems.

**Conclusion:**

Even in severe hemorrhagic episodes during LDLT for BCS, successful outcomes can be ensured by multidisciplinary coordination, advanced monitoring, and adherence to transfusion protocols.

## INTRODUCTION

Hepatic congestion, hepatomegaly, and progressive liver dysfunction are the results of Budd-Chiari Syndrome (BCS), an uncommon hepatic condition brought on by obstruction of hepatic venous outflow.^1^,^2^ The prevalence is approximately one per 100,000 worldwide; however, higher rates have been reported in some Asian countries, such as China, India, and Nepal, while the prevalence in Western populations is significantly lower.^3^,^4^ Idiopathic cases are still very rare.^5^

Etiologic patterns vary geographically, with prothrombotic and inflammatory conditions more frequently implicated in South and East Asia.^6^ Clinically, BCS presents along a spectrum from asymptomatic congestion to fulminant hepatic failure, most often with signs of portal hypertension such as ascites, splenomegaly, and variceal bleeding. Early detection and intervention are essential to prevent irreversible hepatic injury.^7^

For advanced or refractory disease, liver transplantation is the definitive treatment. Living donor liver transplantation (LDLT) is particularly important in regions with limited deceased donor availability but remains technically demanding due to distorted venous anatomy, severe portal hypertension, and complex intraoperative hemodynamics.^8^,^9^

Resulting from infection with Echinococcus granulosus, hydatid cysts are endemic in some regions of Pakistan and can occur on occasion with hepatic venous occlusion. It adds further surgical and anesthetic complexity, requiring careful cyst management to prevent anaphylaxis or spread of infection.^10^,^11^

This report describes the successful anesthetic and perioperative management of a 30-year-old woman with BCS complicated by a hepatic hydatid cyst and previous pulmonary tuberculosis who underwent LDLT at the Pakistan Kidney and Liver Institute and Research Center (PKLI & RC), Lahore. The case is presented according to CARE guidelines.^12^

## CASE PRESENTATION

### Patient Information:

An unmarried 30-year-old woman, 42 kg in weight and 160 cm in height (BMI 16 kg/m², underweight), having blood group O positive, was brought to the Pakistan Kidney and Liver Institute and Research Center (PKLI & RC), Lahore, on September 9, 2025 for a scheduled living donor liver transplantation (LDLT). She had been diagnosed with Budd-Chiari Syndrome (BCS) resulting in decompensated chronic liver disease, along with a concurrent hepatic hydatid cyst.

### Primary Concerns and Symptoms:

The patient presented with progressive jaundice, recurrent hematemesis (multiple episodes over the past month), abdominal distension due to ascites, intermittent hepatic encephalopathy (for six months), one episode of melena, and marked unintentional weight loss (3kg over 25 days).

### Medical, Family, and Psychosocial History:

She had a past history of pulmonary tuberculosis 10-12 years back for which she received a complete course of anti-tuberculous treatment. There was no history of known drug allergy, smoking, alcohol, or substance abuse. No family or psychosocial history was significant. The patient was unmarried with no previous pregnancy.

### Relevant Past Interventions and Outcomes:

Two years before, she had a diagnostic laparotomy for an unrelated abdominal pathology with no postoperative complications. She had received two sessions of endoscopic variceal band ligation in 2025 (February and May) for Grade II esophageal varices, which were followed by mucosal sclerosis.

### Physical Examination and Clinical Findings:

Physical Examination and Clinical Findings: In the preoperative evaluation, she was alert and oriented (GCS 15/15), mildly jaundiced with a lean build. Her vital signs were stable; she had blood pressure of 113/80 mmHg, pulse 90/min, oxygen saturation 100% on room air, and temperature 36°C. Examination of the cardiovascular and respiratory systems revealed normal findings. Abdominal examination revealed a soft, mildly distended, non-tender abdomen with an uneventful surgical scar. Neurological examination showed no focal deficits and no peripheral edema. Airway evaluation revealed Mallampati Class II, normal neck range of motion, and good mouth opening.

### Timeline of the Episode of Care:

**Fig.1:**
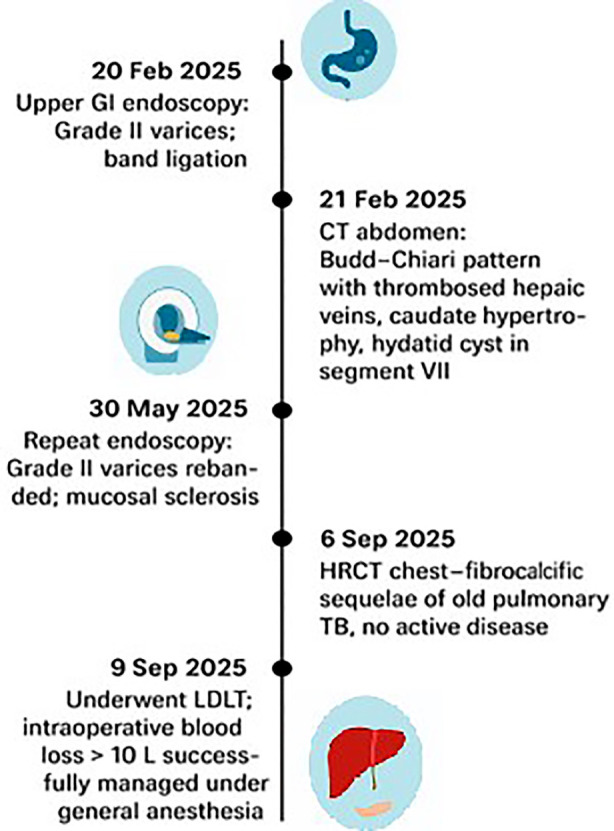
Timeline listing the timeline of diagnostic tests, interventions, and liver transplant events in the Budd-Chiari Syndrome patient.

The temporal progression of the evaluation of the patient, diagnostic results, and surgical treatment is delineated in Fig.1.

### Diagnostic testing:

Preoperative laboratory findings revealed anemia with a mild electrolyte imbalance but otherwise normal renal and coagulation profiles. Table-I shows the comprehensive laboratory parameters and disease severity scores.

**Table-I:** Preoperative Laboratory and Disease Severity Parameters of the Recipient.

Parameter	Result	Normal Range	Interpretation
Hemoglobin (Hb)	7.6 g/dL	12-16 g/dL	Low (anemia)
Total Leukocyte Count (TLC)	6.14 × 109/L	4.0-11.0 × 109/L	Normal
Platelet Count	265 × 109/L	150-400 × 109/L	Normal
Serum Sodium (Na)	133 mmol/L	135-145 mmol/L	Mild hyponatremia
Serum Potassium (K)	3.25 mmol/L	3.5-5.0 mmol/L	Mild hypokalemia
Serum Creatinine	0.72 mg/dL	0.6-1.2 mg/dL	Normal renal function
Total Bilirubin	1.76 mg/dL	0.2-1.2 mg/dL	Mildly elevated
Serum Albumin	3.77 g/dL	3.5-5.0 g/dL	Near normal
INR	1.04	0.8-1.2	Normal
aPTT	29 seconds	25-35 seconds	Normal
Fibrinogen	357 mg/dL	200-400 mg/dL	Normal
MELD Score	14	—	Moderate disease severity
Child-Turcotte-Pugh (CTP) Class	A6	—	Well-compensated liver disease

Interpretation: The laboratory profile reflected maintained hepatic synthetic capacity despite chronic hepatic venous congestion.

**Fig.2:**
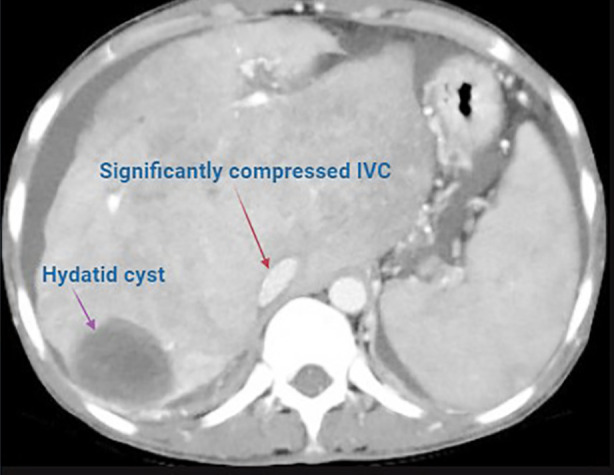
Preoperative Triphasic CT Abdomen Showing Features of Chronic Budd-Chiari Syndrome. Triphasic CT abdomen demonstrating heterogeneous hepatic parenchymal enhancement with caudate lobe hypertrophy, thrombosed right hepatic vein, and non-visualized middle and left hepatic veins. Other findings are moderate-to-severe ascites, segment VII hydatid cyst, tiny hypervascular regenerative nodules in the right lobe, and an eccentric bland thrombus spanning the right portal vein to the main portal vein up to the junction of the portomesenteric. A small thrombus is also seen in the right renal vein near its drainage into the inferior vena cava.

Preoperative CT abdomen showed typical “nutmeg liver” appearance compatible with Budd-Chiari Syndrome with thrombosed hepatic veins, hypertrophied caudate lobe, splenomegaly, moderate ascites, and a discrete 5.8 × 4 cm hydatid cyst in hepatic segment VII (Fig.2).

High-resolution CT (HRCT) of the chest showed evidence of old healed granulomatous disease, with no signs of active pulmonary infection. With an ejection fraction of 62% and no valvular dysfunction, transthoracic echocardiography revealed normal left ventricular systolic performance. A moderate restrictive ventilatory defect was found by spirometry, most likely as a result of ascites and inanition. The absence of systemic or intra-abdominal infection prior to transplantation was confirmed by microbiologic tests, such as blood and ascitic fluid cultures, which showed no signs of bacterial growth. The main challenge in diagnosis involved separating hepatic dysfunction from venous outflow obstruction and intrinsic parenchymal failure. The team needed to confirm that pulmonary tuberculosis had fully resolved before performing transplant surgery. The patient received organized multidisciplinary optimization which made her suitable for surgery despite her low BMI and poor nutritional condition.

### Diagnosis

The patient received a final diagnosis of Budd-Chiari Syndrome because she had a hydatid liver cyst and chronic liver disease that caused decompensation through intermittent hepatic encephalopathy and repeated variceal bleeding. The patient presented with a MELD score of 14 and CTP score of A6 which showed she maintained her synthetic function but displayed clinical decompensation. This apparent disparity is typical of BuddChiari Syndrome, in which relatively stable biochemical. These can also be accompanied by severe complications associated with portal hypertension. Because of the recurrent upper gastrointestinal bleeding and encephalopathy, her prognosis was uncertain otherwise than by transplantation. Her prognosis was good after successful LDLT, provided that postoperative complications were under control and the graft function remained stable.

### Types of Therapeutic Intervention:

- ***Surgical:*** Living donor liver transplantation (recipient).
- ***Pharmacologic:*** General anesthesia, massive transfusion management, perioperative antibiotics, and vasoactive support.
- ***Supportive:*** Cell saver autotransfusion, hemodynamic monitoring (PiCCO- and TEE-guided), goal-directed fluid therapy, and postoperative ICU care.

### Intraoperative Management:

General anesthesia was induced using midazolam, fentanyl, lignocaine, propofol, and cisatracurium and was followed by endotracheal intubation. The anesthesia was maintained using oxygen and isoflurane at MAC 1.0-1.5. Ventilation settings consisted of a tidal volume of 8 mL/kg and PEEP of 3 cm H_2_O. Anesthetic management details are provided in Table-II.

**Table-II:** Intraoperative Anesthetic Management During Living Donor Liver Transplantation.

Phase / Component	Medication / Setting	Dose / Details	Remarks
Induction agents	Midazolam	0.1 mg/kg intravenous	Sedation and anxiolysis
	Fentanyl	2 µg/kg intravenous	Analgesia; blunts intubation response
	Lignocaine	1.5 mg/kg intravenous	Attenuation of airway reflexes
	Propofol	2 mg/kg intravenous	Hypnotic induction
	Cisatracurium	0.2 mg/kg intravenous	Neuromuscular blockade for intubation
Airway management	Endotracheal tube	7.0 mm internal diameter, fixed at 20 cm	Bilateral air entry confirmed
Maintenance	Oxygen + isoflurane	MAC 1.0-1.5	Depth of anesthesia titrated
	Ventilation settings	Tidal volume 8 mL/kg, PEEP 3 cm H₂O	Lung-protective strategy

Right radial arterial line and right internal jugular central venous catheter were inserted for invasive monitoring and vasoactive agent administration. A left femoral PiCCO line was used for enhanced hemodynamic monitoring. Other access included a 9 Fr vascular sheath for transfusion, Foley catheter (16 Fr) for urinary output, nasogastric tube (16 Fr) for decompression, fluid manipulant was done with or without volume estimation using intraoperative TEE. The patient was given 200 mL of albumin, 12 g of mannitol (20%), and 500 mg of methyl prednisolone intravenously during the surgery. Also, broad-spectrum antibiotic prophylaxis included three intravenous doses of piperacillin-tazobactam (4.5 g), one dose of meropenem (1 g), and 200 mg of fluconazole (IV) for antifungal prophylaxis. These medications were given as part of the institutional liver transplant guidelines for maintaining intraoperative fluid management and infection prevention measures.

**Fig.3:**
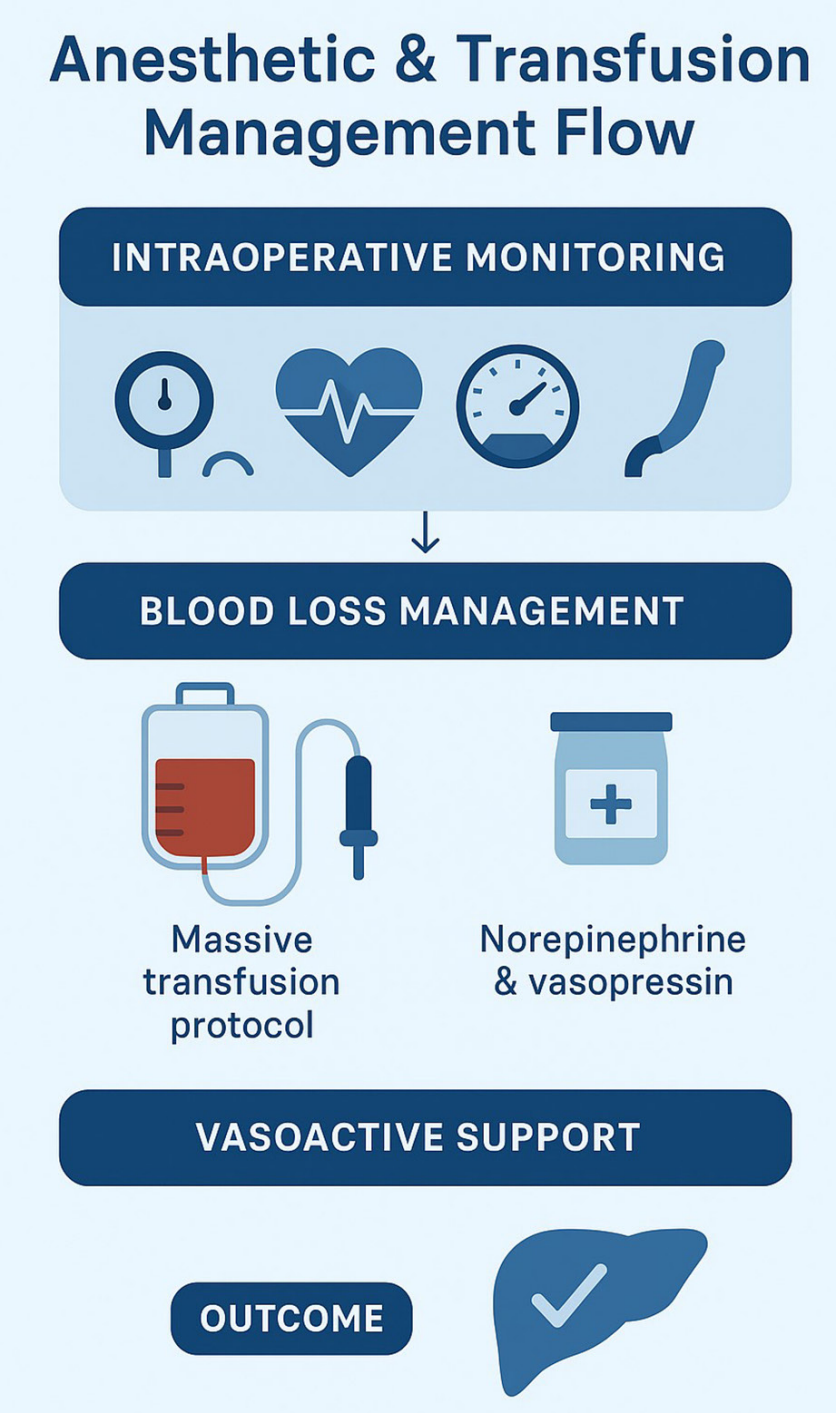
Intraoperative Anesthetic and Transfusion Management Flow during Living Donor Liver Transplantation in Budd-Chiari Syndrome.

To keep mean arterial pressure above 70 mmHg, continuous infusions of norepinephrine (0.16 μg/kg/min) and vasopressin (2.4 IU/h) were titrated based on TEE results and PiCCO parameters. Intraoperative fluid resuscitation was performed using one liter of isotonic crystalloid solution, 200 milliliters of 5% albumin, and 8.8 liters of Ringer’s lactate. Six units of packed red blood cells, four units of fresh frozen plasma, and four units of cryoprecipitate were used to replace blood products. Furthermore, a cell saver system was used to reinfuse 2.9 litres of autologous blood. Approximately 10 litres of blood were lost during the procedure, and 700 millilitres of urine were produced overall. A positive 500 mL net intraoperative fluid balance allowed for adequate tissue perfusion without leading to fluid overload. Goal-directed transfusion and careful monitoring maintained haemodynamic stability despite an estimated blood loss of over 10 litres. Haemodynamic stability, blood conservation, and early transfusion techniques were prioritized in well-coordinated intraoperative care. Haemodynamic stability, blood conservation, and early transfusion measures were prioritized in the well-coordinated intraoperative care (Fig.3).

### Changes in Therapeutic Intervention:

Massive bleeding during dissection during surgery set off the massive transfusion protocol and cell saver autotransfusion. Based on PiCCO-derived values and TEE results, haemodynamic management was switched to goal-directed therapy. ROTEM was used to monitor coagulation, and following component therapy, it was discovered to be within normal bounds.

### Clinician and Patient-Assessed Outcomes:

Throughout the 14 hours procedure, the patient’s haemodynamics remained stable. The MAP (mean arterial pressure) was maintained at or above 70 mmHg. There were no events during reperfusion. Haematocrit was 31% and intraoperative final haemoglobin was 10.8 g/dL. With stable vital signs, she was moved to the surgical intensive care unit (ICU) on low-dose norepinephrine (0.16 µg/kg/min) and vasopressin (2.4 IU/h).

### Follow-up outcomes:

**Fig.4:**
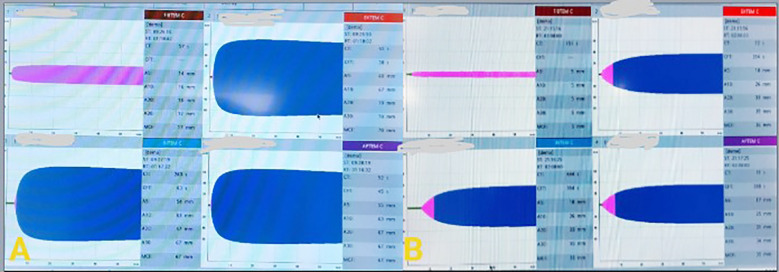
ROTEM assessment of coagulation parameters. A. ROTEM at the beginning of surgery B. ROTEM at the conclusion of surgery

Haemoglobin of 10.8 g/dL and lactate of 6.9 mmol/L were found during intraoperative investigations; these values peaked at 7.1 mmol/L during surgery but continued to show a declining trend in the postoperative period, indicating improved tissue perfusion. Focused component therapy also restored the ROTEM-based coagulation markers to normal ranges, indicating adequate intraoperative coagulopathy restoration (Fig.4). During the early stages of recovery, the patient’s electrolyte and renal status remained unchanged. Serial Doppler ultrasonography scans were performed to confirm adequate graft perfusion and hepatic vasculature patency in order to rule out biliary blockage or vascular impairment. There were no intraoperative cardiac or pulmonary problems, and well-tolerated interventions included extended anesthetic and large-volume transfusion. The resuscitation and anesthetic processes were completed on time. The institutional large transfusion protocol and the liver transplant anesthetic policy were also well followed.

### Postoperative Outcomes:

The patient was extubated in the surgical intensive care unit due to stable hemodynamics and adequate graft function. Good graft perfusion and patent hepatic vasculature were verified by serial Doppler ultrasounds. Laboratory results showed that coagulopathy was being corrected and liver function tests were continuously improving. Following surgery, there were no vascular, biliary, or complication as infections during the hospital stay. The duration of hospital stay was 20 days. The patient was discharged stable with adequate graft function and was advised to follow regular follow-up appointments at the transplant clinic.

## METHODOLOGY

### Literature Search Strategy:

A targeted review of the literature was performed using PubMed, Google Scholar, and Scopus to find publications between 2015 and 2025 with the search terms “Budd-Chiari Syndrome,” “massive transfusion,” “living donor liver transplantation,” and “hydatid cyst.” English-language case series and reports only including perioperative or anesthetic care in liver transplantation were selected, and relevant data were integrated into the discussion.

### CARE Checklist (2013) Statement:

The authors have read the CARE Checklist (2013), and the manuscript was prepared and revised in accordance with the CARE Checklist guidelines.

## DISCUSSION

Anesthetic care of a Budd-Chiari Syndrome (BCS) patient who underwent living donor liver transplantation (LDLT) complicated with a hepatic hydatid cyst and more than ten-liter intraoperative bleeding is an extremely complicated clinical scenario. Even though liver transplantation due to BCS has been documented extensively, comprehensive reports of anesthetic management during such severe bleeding episodes are scarce. Due to challenging surgical anatomy and extensive collateral circulation, this case emphasises the contrast between a relatively stable preoperative status (MELD 14, CTP A6) and the risk of severe intraoperative haemodynamic instability. By contrasting the perioperative care in this instance with data from previously published series, Table-III contextualizes our findings and highlights the key elements that contribute to a successful outcome given a substantial intraoperative challenge.

**Table-III:** Comparative Analysis of Anesthetic and Perioperative Management in Budd-Chiari Syndrome Patients Undergoing Liver Transplantation.

Feature	Present Case	Comparative Literature Findings	Key Implications for Management
***Patient Profile***			
Age / Sex / BMI	30 y / F / 16 kg/m² (Low BMI)	Range: 19-66 yrs; male predominance. BMI often unreported.	Low BMI poses anesthetic and nutritional challenges affecting dosing, fluid tolerance, and recovery.
Disease Severity	CTP A6, MELD 14 (Well-compensated)	Obed et al.^13^: Mean MELD 32; Doğrul et al.^14^: Mean MELD 18; Segev et al.^15^: Broad range, mostly higher scores.	Significant blood loss can occur even in low-MELD cases; surgical complexity, not physiologic reserve, often dictates risk.
Comorbidities	Hepatic hydatid cyst, prior tuberculosis	Rocha-Santos et al.^16^: HCC; Tekbaş et al.^17^: MPN; other prothrombotic states (Factor V Leiden, Protein C/S deficiency).	Hydatid cysts increase surgical complexity and risk of anaphylaxis or rupture.
***Surgical & Anesthetic Setup***			
Transplant Type	Living donor (LDLT)	Mixed: LDLT in Rocha-Santos, Doğrul, Obed; DDLT predominant in Segev.^13^-^17^	LDLT is often the only option in regions with limited DDLT access and requires advanced venous reconstruction.
Monitoring	Arterial, CVP, PiCCO, TEE	Brezeanu et al.^18^: Advocates PiCCO, TEE, and ROTEM as crucial. Obed et al.^13^: Highlights vascular challenges.	Validates the role of multimodal monitoring for precise goal-directed therapy.
***Intraoperative Course***			
Estimated Blood Loss	~10 L (massive)	Typically underreported; Rahman et al.^19^: 3 units PRBC; Obed et al.^13^: 0-6 units PRBC.	Represents an extreme of hemorrhage; underscores importance of robust transfusion strategy.
Transfusion Strategy	MTP: 6 PRBC, 4 FFP, 4 Cryo + 2.9 L Cell Saver	Ratios seldom detailed; Brezeanu et al.^18^: ROTEM-guided therapy recommended.	Demonstrates efficacy of balanced transfusion with autologous salvage for stability and conservation.
Vasopressor Support	Norepinephrine + Vasopressin	Common practice though variably detailed.	Dual-agent therapy is essential to maintain perfusion under extreme vasodilation.
***Outcomes***			
Hemodynamic Stability	Maintained MAP >70 mmHg	Target consistent across series but difficult to sustain.	Reinforces the value of aggressive monitoring and protocolized support.
Reperfusion Syndrome	None	Brezeanu et al.^18^: Common but preventable with optimization.	Suggests that pre-reperfusion correction of acidosis, electrolytes, and volume prevents PRS.
Graft Function / Survival	Stable graft; uneventful recovery	Doğrul et al.: 1/3/5-yr survival 87/71/71%; Obed et al.: 84/67/67%; Segev et al.: ~85% at 3 yrs.^13^-^15^	Outcomes comparable to international benchmarks, reflecting high-quality perioperative care.

***Note:*** PRBC: packed red blood cells

As shown in Table-III, our patient’s MELD score was lower than what is typically reported; patients with Budd-Chiari Syndrome would be considered to have advanced liver failure.^13^–^14^ Because the complexity of the procedure would depend more on factors like the degree of venous obstruction, the development of collateral vessels, and the coexistence of conditions, this suggests that prior prognostic scores may not always be a reliable indicator of intraoperative risk in BCS. An unanticipated hepatic hydatid cyst in BCS increased the risk of bleeding and made dissection more challenging. These required close collaboration between anaesthesia and surgical staff as well as careful anaesthetic control.

In this instance, invasive haemodynamic monitoring guided the administration of anaesthesia. Although TEE and PiCCO are ideally suited for complex liver transplants, our experience indicates that they can be employed to control intraoperative profound instability. TEE provided immediate information on ventricular filling and cardiac function, while PiCCO enabled continuous monitoring of cardiac output and vascular resistance, which guided fluid resuscitation and transfusion. These monitors allowed for customized norepinephrine and vasopressin titration to keep the mean arterial pressure at 70 mmHg and, consequently, adequate graft perfusion despite the significant bleeding. The experience also shows that, even in a context with limited resources, a methodical transfusion strategy and cautious use of high-tech surveillance may provide results comparable to those from larger overseas centers. The most difficult intraoperative experience was managing almost 10 liters of blood loss much more than has ever been reported in situations like this. This incident highlights the unpredictable risk of bleeding linked to Budd Chiari Syndrome and the significance of an active Massive Transfusion Protocol in directing a planned resuscitation. Six units of packed red blood cells, four units of fresh frozen plasma, and four units of cryoprecipitate were used in component balanced therapy to preserve coagulation and hemodynamic stability.

While FIBTEM A10 guided the delivery of cryoprecipitate, ROTEM-guided transfusion was based on EXTEM CT and EXTEM A10 to determine the requirement for fresh frozen plasma. According to our institutional protocol, the objective values were an EXTEM CT < 80–90 seconds, an EXTEM A10 > 40–45 mm, and a FIBTEM A10 > 8–10 mm. Reinfusing 2.9 liters of autologous blood with cell salvage also decreased the demand for donor blood. These results show that including cell salvage into an MTP protocol for the liver transplant operation for non-malignant illnesses is both feasible and advantageous.

## CONCLUSION

Acute intraoperative bleeding in LDLT for Budd-Chiari Syndrome is a clinical situation that necessitates thorough preoperative evaluation, close hemodynamic monitoring, and rigorous adherence to a well-defined transfusion regimen in order to effectively control anesthesia. This example shows how a high-risk operation may be turned

### Patient perspective:

The patient expressed satisfaction with the procedure’s overall care and results. Throughout her therapy, she was happy with the interdisciplinary approach and timely communication. The patient reported that after recovering, her quality of life and symptoms greatly improved.

### Informed consent:

Informed written consent was taken from the patient for the publication of this case report and accompanying images. Publication ethical approval was obtained from the Pakistan Kidney and Liver Institute and Research Center (PKLI & RC), Lahore Institutional Review Board (IRB) (IRB Reference No: PKLI-IRB/AP/00982025).

### Authors’ Contribution:

**SH, EUDK, SS:** Concept and Design, Data Collection, Manuscript Drafting, Critical Revision of Manuscript,

**YBB:** Data Collection, Concept and Design, Critical, Revision of Manuscript,

**FA:** Data Collection, Critical Revision of Manuscript,

All authors have read and approved the final version and are also accountable le for the integrity of the study.
